# Blocking CD47 Shows Superior Anti-tumor Therapeutic Effects of Bevacizumab in Gastric Cancer

**DOI:** 10.3389/fphar.2022.880139

**Published:** 2022-05-25

**Authors:** Chenyang Shi, Jiaxin Li, Guorong Fan, Yu Liu

**Affiliations:** ^1^ Department of Clinical Pharmacy, Shanghai General Hospital, Shanghai Jiao Tong University School of Medicine, Shanghai, China; ^2^ Department of Biochemistry, School of Life Science and Technology, China Pharmaceutical University, Nanjing, China

**Keywords:** VEGF-A, CD47, antiangiogenesis, immunosuppressive, synergism

## Abstract

**Background**: Bevacizumab (Avastin^®^), a humanized antiangiogenic monoclonal antibody, is widely used in the clinical treatment of tumour diseases. However, recent research has shown that the beneficial antiangiogenic effects of these agents have been limited in a number of patients due to complex immunosuppressive mechanisms. Here, we report a synergistic antitumour strategy through simultaneous blockade of VEGF and CD47 signalling to enhance the curative effect of advanced gastric cancer.

**Method**: A BGC-823 gastric tumour model was chosen to evaluate antitumour efficacy. Macrophage migration and phagocytosis were evaluated to determine immune-related resistance to bevacizumab therapy. Synergistic antitumour activity was observed on the basis of tumour volume, tumour weight, tumour inhibition rate, tumour angiogenesis and tumour metastasis when bevacizumab was combined with an anti-CD47 monoclonal antibody.

**Results**: Our study demonstrated that synergistic therapy targeting CD47 and VEGF reversed macrophage migration and phagocytosis, which were inhibited by antiangiogenic therapy and enhanced antitumour effects. Moreover, blockade of CD47 induced by antiangiogenic therapy inhibited tumour metastasis.

**Conclusion**: Our data provide an effective strategy to attenuate resistance to bevacizumab therapy, promoting clinical cancer treatment with antiangiogenic drugs in combination with CD47-targeting inhibitors.

## Introduction

Angiogenesis is an important hallmark for the progression of solid tumours. A series of factors have been considered as regulators of angiogenesis, and vascular endothelial growth factor (VEGF) has attracted considerable attention in the process of tumour growth and metastasis (Melincovici et al., 2018). The humanized anti-human VEGF-A monoclonal antibody bevacizumab (Avastin^®^), approved by the Federal Food and Drug Administration (FDA) in 2004, has been widely used in the clinical treatment of several malignant diseases (Minion and Tewari, 2017; Garcia et al., 2020). Anti-VEGF is a cancer therapy that inhibits endothelial cell proliferation and new vessel growth; however, its beneficial effects have been limited in a number of patients (Huijbers et al., 2016; Itatani et al., 2018; Haibe et al., 2020). An anti-VEGF strategy was recently shown to trigger tumour relapse and metastasis, and a plausible relapse mechanism suggests that the effect of anti-VEGF may lead to immunosuppression (Tamura et al., 2019; Zhang et al., 2019; Song et al., 2020). In addition, VEGF-A can trigger tumour-induced regulatory T-cell proliferation, inhibit the maturation of dendritic cells, and contribute to the state of immune suppression (Terme et al., 2013; Hodi et al., 2014).

The inhibition of tumour angiogenesis could paradoxically lead to hypoxia tolerance of cancer cells, especially in the centre of the tumour mass (Tian et al., 2020). Studies of anti-VEGF resistance have revealed that hypoxia is a key factor in the development of an immunosuppressive microenvironment (Croci et al., 2018). Hypoxia-inducible Factor 1 (HIF-1) plays an indispensable role in the immune evasion of hypoxic tumours. Zhang et al. found that HIFs stimulate the production of CD47 (cluster of differentiation 47), a protein on the tumour cell surface that transmits “don’t eat me” signals to avoid destruction by macrophages, via interaction with the signal regulatory protein (SIRP)-α expressed on the surface of macrophages (Zhang et al., 2015). Moreover, there is growing evidence that the CD47-SIRPα axis may also inhibit antigen-presenting cell function and thereby suppress adaptive T cell-mediated anticancer immunity (Matlung et al., 2017). It has been shown that CD47 overexpression in most solid tumours, including breast cancer, non-small cell lung cancer and gastric cancer, is associated with poor prognosis. Several clinical trials have demonstrated that blockade of the CD47/SIRPα pathway is an effective treatment for solid tumours and haematologic malignancies (Zhang et al., 2020).

Based on the above mechanisms, bevacizumab, the humanized anti-human VEGF-A monoclonal antibody, was combined with B6.H12, an anti-CD47 antibody, to assesse the anti-tumor effects of VEGF/CD47 dual targeting therapy. In this study, we investigated the macrophage migration and phagocytosis to determine immune-related resistance to anti-VEGF therapy *in vitro*. Furthermore, the anti-tumor effcacy of cotreatment with bevacizumab and anti-CD47 antibody has been demonstrated in BGC-823 gastric xenograft mouse model. We report here on the antitumour efficacy with blockade upregulating CD47 expression in the tumour microenvironment and conferring gastric cancer resistance to antiangiogenic therapy.

## Material and Methods

### Reagents

The antibodies and reagents were obtained as follows: the B6H12 monoclonal antibody that reacts with human CD47 (Bio X Cell, Lebanon NH, United States) and bevacizumab (Roche Genentech, South San Francisco, United States); PE anti-CD11b antibody and Alexa Fluor^®^594 anti-CD68 antibody (Abcam, Cambridge, United Kingdom); HiScript II 1st Strand cDNA Synthesis Kit and AceQ Universal SYBR qPCR Master Mix (Vazyme, Nanjing China).

### Cell Lines and Cell Culture

The human gastric cancer cell lines SGC-7901, BGC-823, SGC-7901-GFP and BGC-823-GFP were generous gifts from the laboratory of Professor Liu Yu (China Pharmaceutical University, China). These cells were cultivated at 37°C in a 5% CO_2_ incubator and cultured in RPMI 1640 (Gibco, San Diego, United States) with 10% FBS (Gibco, San Diego, United States).

### Experimental Animals

BALB/c nude mice (18–22 g) were purchased from the Experimental Animal Center of Yangzhou University (Yangzhou, China). All mouse-related experiments were approved by the Institutional Review Committee for the use of Human or Animal Subjects.

### In Vitro Phagocytosis Assay

Macrophage phagocytosis was detected as described previously. Briefly, primary mouse macrophages were obtained from femurs of BALB/c nude mice and then incubated with medium containing macrophage colony-stimulating factor (MCSF 20 ng/ml) for 7 days. Macrophages were labelled with PE anti-CD11b antibody and cocultured with SGC-7901-GFP or BGC-823-GFP cells at 37°C for 4 h. After treatment, the cells were washed three times with PBS and analysed via flow cytometry (BD Biosciences, San Jose, CA, United States). The phagocytosis index is presented as the ratio of GFP + phagocytic macrophages to total macrophages.

### In Vitro Migration Assay

The migration of macrophages was studied via coculture of macrophages and BGC-823 cells using Transwell chambers with 8-µm pores (Corning, Corning, NY, United States) in a 24-well plate. One hundred microlitres (5 × 10^5^ cells/ml) of macrophages and 500 μl (2 × 10^5^ cells/ml) of BGC-823 cells were cultured in the upper and lower compartments of a Transwell chamber, respectively, and allowed to migrate at 37°C in 5% CO_2_. After coculture with treatment (Bev 10 μg/ml, anti-CD47 10 μg/ml or Bev 10 μg/ml + anti-CD47 10 μg/ml) for 24 h, the nonmigrating cells in the upper chambers were cleaned with a cotton swab and then stained with 0.1% crystal violet solution for 60 min. Migratory cells were imaged using an inverted microscope (Motic, Xiamen, China), and the results of cells per well were evaluated in 3 high-power fields.

### In Vivo Xenograft Tumour Model

BGC-823-GFP cells (2×10^6^) were injected subcutaneously into the right flanks of 4 to 6-week-old BALB/c nude mice to establish a xenograft model. When the tumours reached 100–300 mm^3^, the tumours were removed and sectioned into 1–2 mm^3^ blocks for an additional mouse inoculation subcutaneously. After 5 days, all well-established mice were randomly assigned to six groups (n = 6): the PBS, bevacizumab (Bev, 10 mg/kg), anti-CD47 antibody (anti-CD47, 10 mg/kg), Bev + anti-CD47 (5 mg/kg), Bev + anti-CD47 (10 mg/kg) and Bev + anti-CD47 (20 mg/kg) group. All administration groups were injected intraperitoneally once per week for 28 days.

### RNA Extraction and qRT-PCR Analysis

Total RNA was extracted from the BGC-823 tumour tissues using TRIzol in accordance with the manufacturer’s instructions. RNA concentration was determined using a Quickdrop (Molecular Devices, CA, United States). A cDNA library was constructed using the HiScript II 1st Strand cDNA Synthesis Kit (Vazyme, Nanjing, China). Quantitative PCR (qPCR) was performed to evaluate mRNA expression using AceQ universal SYBR qPCR Master Mix (Vazyme, Nanjing, China). The qPCR conditions were as follows: predenaturation at 95°C for 5 min and 40 cycles of amplification at 95°C for 60 s and 60°C for 30 s.

### Histology, Immunohistochemistry and Immunofluorescence Staining

The tumors were isolated and fixed with 4% formaldehyde, and then, paraffifinembedded 4-μm tissue sections were processed. The sections were deparaffinized and rehydrated before detection. For histological observation, tumor sections were stained with hematoxylin and eosin (H and E), and then observed by an inverted microscope (Motic, Xiamen, China).

For immunohistochemistry analysis, prior to the application of the primary antibody, normal goat serum was incubated to block the nonspecifific protein binding sites for 10 min. Then, a rabbit anti-CD31 polyclonal antibody was incubated as the primary antibodies for detecting CD31. After washing with PBS, the slides were treated for 1.5 h with secondary antibody at room temperature. After a 15-min incubation at 37°C, the slides were washed with PBS and incubated with diaminobenzidine (DAB) chromogen for 3–5 min to yield a dark brown color. The sections were counter-stained with hematoxylin for microscopic observation.

CD68 antibodies are used in current practice as markers for monocytes/macrophages present in normal or pathological tissue fixed in paraffin. For immunofluorescence staining, serial sections of the same specimens were incubated with Alexa Fluor^®^594 anti-CD68. All images were imaged using an inverted fluorescence microscope (Zeiss, Germany).

### Metastasis Analysis

For metastasis studies in mice receiving subcutaneous BGC-823-GFP cells, the mice were euthanized and lungs collected after antitumor test on the last day. The lungs were fixed in 10% neutral buffered formalin at 4°C followed by dehydration. Sections (10-μm thickness) were processed with a microtome and observed using an inverted fluorescence microscope (Zeiss, Germany).

### Statistical Analysis

The data of our work were analysed by GraphPad Prism7 and Origin 8. The statistical significance of a difference was determined by Student’s t-test. A *p* value below 0.05 was regarded as statistically significant, and ns was considered a nonsignificant difference (*p* > 0.05).

## Results

### Inhibiting CD47 Promotes Macrophage Migration and Phagocytosis

**FIGURE 1:**
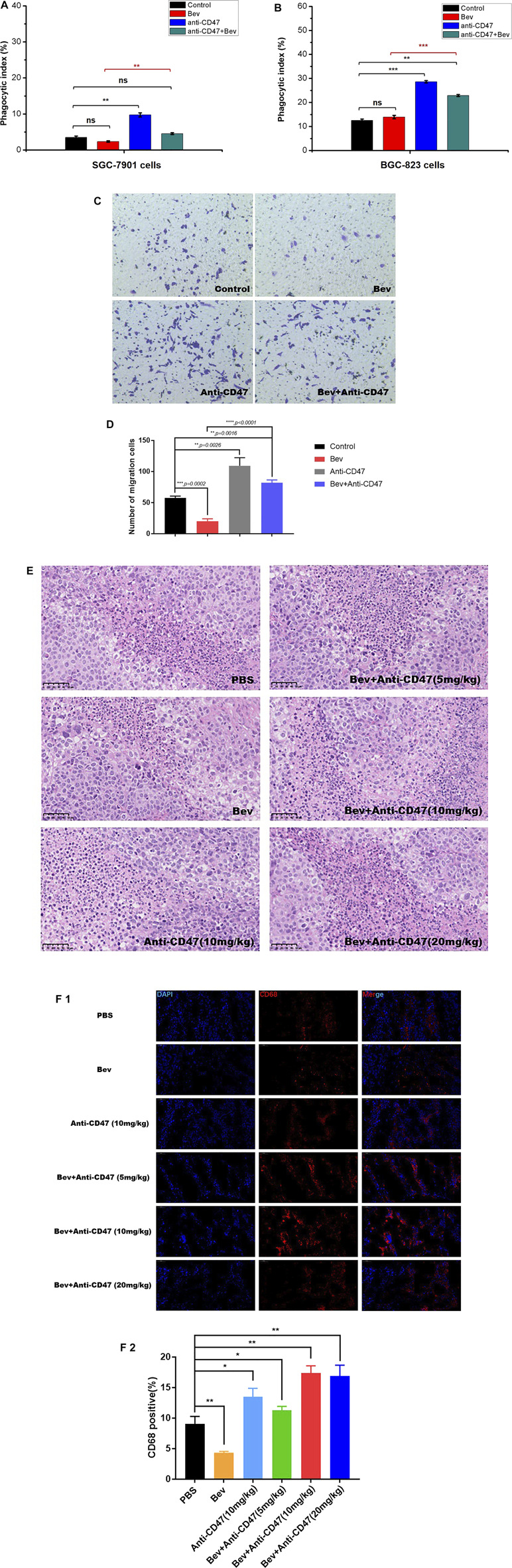
Targeting CD47 reverses macrophage phagocytosis and migration to gastric cancer cells, reducing antiangiogenic therapy. **(A)**, **(B)** Phagocytic index indicating the phagocytosis of macrophages against gastric cells mediated with monotherapy or combination therapy (A SGC-7901 cells, B BGC-823 cells, n = 5). **(C)**, **(D)** BGC-823 cells and macrophages were cultured in Transwell chambers with monotherapy or combination therapy. Migrated cells were stained with crystal violet and counted. Magnification was 200x. **(E)** H&E staining of monocyte infiltration in each group, scale, 50 μm. **(F)** Fluorescence staining of CD68 to detect macrophage depletion, scale, 50 μm. All the data are presented as the mean ± SD (*p < 0.05, **p < 0.01, ***p < 0.001, and ****p < 0.0001).

We designed *in vitro* macrophage migration and phagocytosis assays to evaluate the efficacy of anti-CD47 mAb alone or with bevacizumab as a therapeutic in gastric cancer, especially for preventing early seeding events. Compared with isotype control IgG1, bevacizumab showed negligible effects on phagocytosis in gastric cancer cells. Treatment with an anti-CD47 mAb alone or with bevacizumab significantly increased the phagocytic index. Flow cytometry showed that 9.75 and 4.54% of the macrophages engulfed cancer cells in the anti-CD47 mAb-alone and bevacizumab treatment, respectively, for SGC-7901 cells, and for BGC-823 cells, the percentages were 28.63 and 22.90%, respectively, which was much higher than that of the bevacizumab group or the IgG1 isotype group. Similar results were also observed for macrophage migration (Figures 1A–D; Supplementary Figures S1, S2; Supplementary Tables S1, S2).

Furthermore, to detect the relevance of monocytes or macrophages and CD47 *in vivo*, Alexa Fluor^®^594 anti-CD68 antibody and HE staining was performed to detect macrophages in the gastric tumour xenograft model (Figures 1E,F; Supplementary Table S3). Compared to the control group, more monocytes or macrophages (CD68 + cells) were observed following anti-CD47 treatment; however, fewer monocytes or macrophages were observed following bevacizumab treatment.

### Bevacizumab Treatment Induces Tumour Cells Expressing CD47 and HIF-1

**FIGURE 2:**
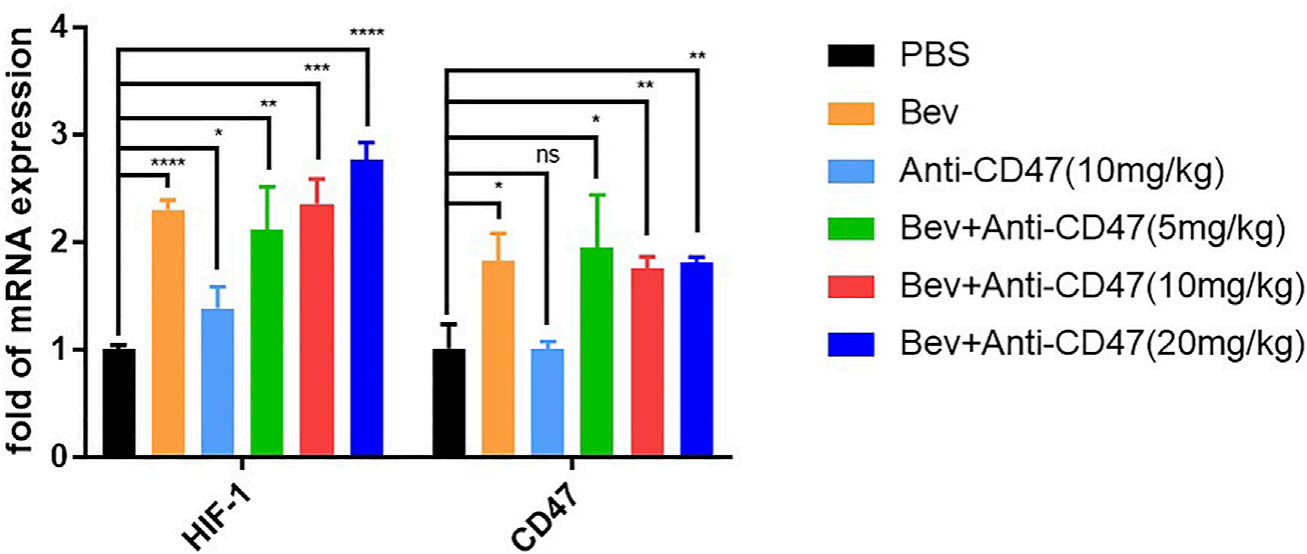
Blocking VEGF upregulate HIF-1 and CD47 mRNA. qPCR results of mouse tumours in each treatment group. All the data were presented as the mean ± SD. Compared with the PBS group: *p < 0.05, **p < 0.01, ***p < 0.001, ****p < 0.0001; n = 3.

We considered whether bevacizumab can upregulate HIF-1 and CD47 expression. The mRNA levels of BGC-823 tumour cells are shown in (Figure 2; Supplementary Table S4). After treatment of gastric cancer xenograft models, the bevacizumab group exhibited upregulation of HIF-1 and CD47 mRNA. Similar results were also demonstrated in anti-CD47 monotherapy or combination therapies.

### Cotreatment With Bevacizumab and anti-CD47 Antibody Demonstrates a Better Antitumour Effect in Gastric Tumour Models

**FIGURE 3:**
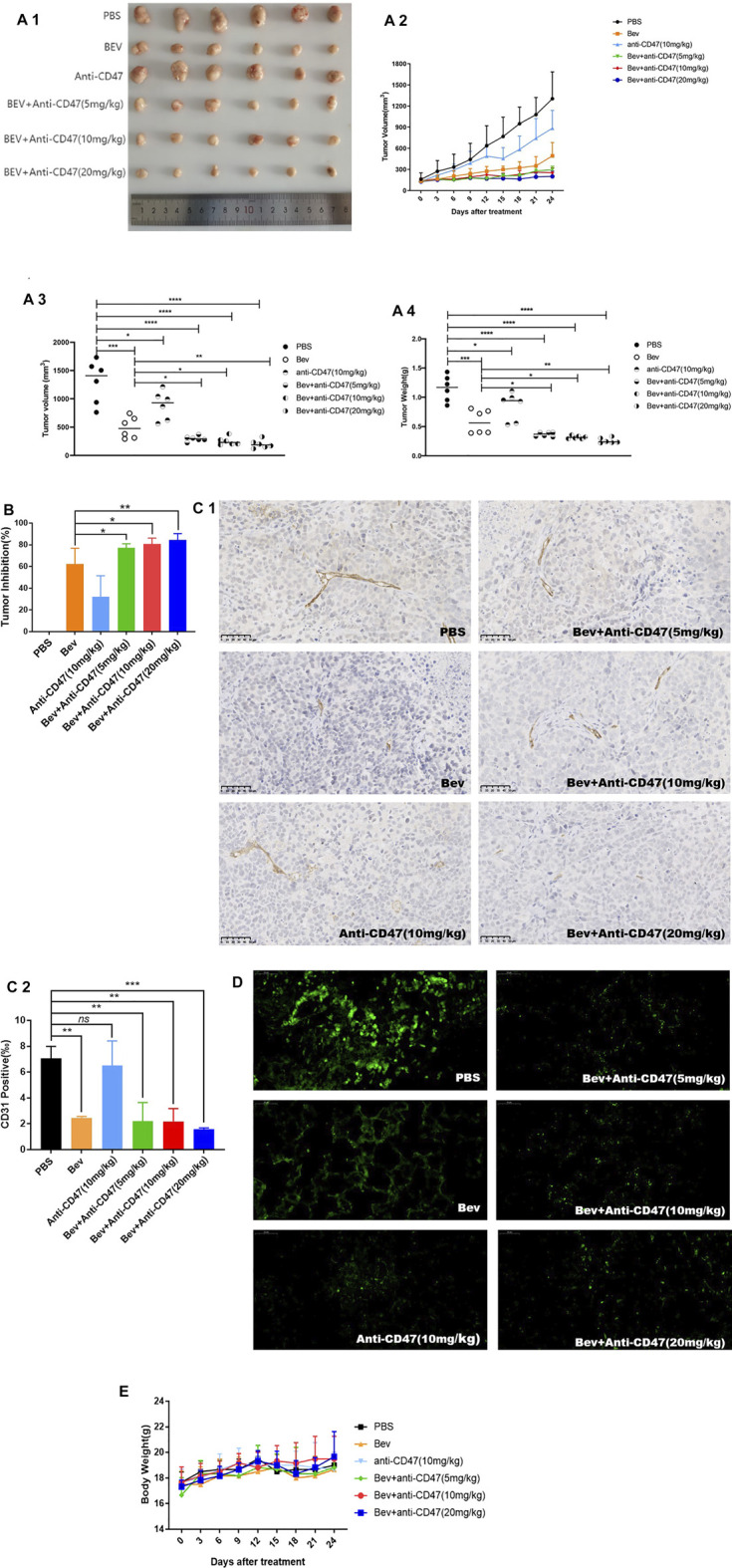
Cotargeting CD47 and VEGF elicited synergetic antitumour effects in gastric cancer. **(A)** In the BGC-823 xenograft model, tumour volume and tumour weight are presented (mean ± SD, N = 6 per group). **(B)** Tumour inhibition was calculated. **(C)** CD31 immunohistochemistry of tumour sections of BALB/c nude mice after injection for 4 successive administrations. **(D)** Spontaneous pulmonary metastasis of BGC-823 gastric cancer cells was observed in lung tissue. **(E)** The change in the mean animal body weight for BALB/c nude mice bearing BGC-823 xenografts treated with different formulations. All the data are presented as the mean ± SD (*p < 0.05, **p < 0.01, ***p < 0.001, and ****p < 0.0001, ns denotes a statistically nonsignificant difference, *p* ≥ 0.05).

There is no apparent reduction of cell proliferation *in vitro* in our previous work (Supplementary Figure S3). Tumour volume, tumour weight and tumour inhibition rate showed that cotargeting VEGF and CD47 could enhance potent antitumour effects. After administration, the Bev + anti-CD47 treatment formulation produced smaller tumour volumes, especially in the Bev (10 mg/kg) + anti-CD47 (20 mg/kg) group (200.24 ± 68.23 mm^3^), whereas the tumour volumes in the Bev group (492.11 ± 173.67 mm^3^) and anti-CD47 group (884.56 ± 230.58 mm^3^) were smaller than those in the tumour PBS group (1,303.75 ± 347.45 mm^3^) (Figures 3A1–3; Supplementary Table S5). As similar result to tumour volume, the Bev + anti-CD47 treatment formulation demonstrated smaller tumour weight, especially in the Bev (10 mg/kg) + anti-CD47 (20 mg/kg) group (0.26 ± 0.05 g), whereas the tumour weight in the Bev group (0.58 ± 0.20 g) and anti-CD47 group (0.85 ± 0.24 g) were smaller than those in the tumour PBS group (1.15 ± 0.22 g). The result was shown in (Figures 3A–4). Based on these results, treatment with Bev (10 mg/kg) + anti-CD47 (20 mg/kg) reduced the tumour volume by 84.64 ± 5.23% (Figure 3B; Supplementary Table S6). In addition, the microvessel-specific marker CD31 was used to evaluate the degree of tumour angiogenesis, which presents as a rapidly growing tumour. We found that treatment with Bev (10 mg/kg) + anti-CD47 (20 mg/kg) decreased the microvessel density of xenograft tumours to 1.59 ± 0.08‰, compared to that of treatment with PBS group (7.06 ± 0.76‰). We further compare and analyse the difference between Bev and Bev + anti-CD47. We found that treatment with Bev (10 mg/kg) + anti-CD47 (5 mg/kg), Bev (10 mg/kg) + anti-CD47 (10 mg/kg), and Bev (10 mg/kg) + anti-CD47 (20 mg/kg) decreased the microvessel density of xenograft tumours to 2.21 ± 1.17‰, 2.19 ± 0.81‰, and 1.59 ± 0.08‰, respectiveiy, compared to that of treatment with Bev group (2.46 ± 0.09‰). These data are consistent with antitumor activity and suggested that combined with anti-CD47 present greater antiangiogenic activity with an increasing dose dependence, especially in Bev (10 mg/kg) + anti-CD47 (20 mg/kg) group (*p* < 0.001) (Figure 3C; Supplementary Table S7). Furthermore, blockade of CD47 and VEGF by specific Abs inhibited spontaneous pulmonary metastasis of BGC-823 gastric cancer cells *in vivo* (Figure 3D). Safety was monitored by assessing animal behaviour and body weight during administration. In all cases, neither the mean animal body weight nor any other obvious adverse reaction was significantly observed over time (Figure 3E; Supplementary Table S8).

## Discussion

Anti-angiogenic therapy plays a critical role in blocking the blood supply for tumours. Many studies have confirmed that bevacizumab, a recombinant human monoclonal antibody, is an effective treatment for the inhibition of tumour angiogenesis. Nevertheless, negative feedback has been reported to inactivate macrophage phagocytosis and even cause immunosuppression during antiangiogenic therapy. In the present study, we revealed bevacizumab significantly decreased the macrophage phagocytic index and migration cell number *in vitro* and *in vivo*. In addition to inhibiting macrophage recruitment by VEGF, we found the upregulation of CD47 as an innate immunosuppressive mechanism that limited the macrophage antitumour effect of VEGF inhibitors. Simultaneous target of VEGF and CD47 significantly improved anti-tumor efficacy and inhibiting tumour metastasis in gastric tumour-bearing mice, which was most likely mediated through facilitating enhanced macrophage infiltration, confirmed synergistic anti-tumor effect following combined treatment with bevacizumab and anti-CD47 antibody.

Previous studies have reported that VEGF-A can recruit macrophages to sites of inflammation, and activated macrophages are known to express a variety of cytokines and growth factors, including VEGF-A, -C, and -D. Indeed, VEGF-A expression has a positive correlation with the number of macrophages in many tumour tissues (Cursiefen et al., 2004; Lee et al., 2013). C. L *et al.* confirmed that knockdown of VEGFR1 blocks macrophage recruitment into tumour tissue in a clear cell renal cell carcinoma xenograft model, indicating that VEGFR1, which is known to be expressed on monocytes and macrophages, induces macrophage recruitment by the VEGFR1/VEGF-A axis (Li et al., 2011). Here, we sought to determine whether neutralization of VEGF by bevacizumab would inhibit macrophage migration and phagocytosis. Our results from the flow cytometry analyses and transwell array showed that Bevacizumab significantly decreased the macrophage infiltration compared to controls; however, inhibiting CD47 reversed macrophage infiltration to gastric cancer cells, which was reduced by antiangiogenic therapy. Moreover, combined treatment with bevacizumab and anti-CD47 antibody enhanced macrophage activation both *in vitro* and *in vivo*.

However, the mechanism by which CD47 is upregulated in gastric cancer cells relapsing after antiangiogenic therapy is not clear. Previous studies have reported that antiangiogenic agents, such as bevacizumab, used as single-agent therapy have yielded disappointing results. One of the reasons may be due to increased intratumour hypoxia and the mediation of adaptive responses to hypoxic conditions that commonly negatively impact the therapeutic outcome. The effects of bevacizumab on intratumour hypoxia and HIF-1 activity have been demonstrated. Moreover, synergistic therapy may further aggravate the hypoxia in the tumor for superior anti-tumor therapeutic effects. However, emerging studies have reported that HIF-1 directly activates CD47 gene transcription in hypoxic tumour cells. Huimin Zhang *et al.* demonstrated that the antiangiogenic sorafenib induces intratumoural hypoxia that causes HIF-1-dependent induction of breast cancer stem cells and then activates the expression of CD47 for immune evasion (Zhang et al., 2015). Similar to the results presented here, the BGC-823 cell line showed upregulated mRNA expression of HIF-1 and cell-surface CD47 after bevacizumab treatment *in vivo*, suggesting that simultaneous blockade of the angiogenetic axis and CD47 axis significantly improved antitumour efficacy by facilitating macrophage infiltration and producing antitumour potency in experimental gastric tumour models. However, anti-CD47 antibodies were unable to inhibit the expression of CD47 mRNA in solid tumour cell lines.

Highly expressed CD47 in multiple tumour cells inhibits phagocytosis by sending the “don’t eat me” signal. Increasing evidence supports targeting CD47 as a novel innate immune checkpoint to enhance the efficacy of immunotherapy (Matozaki et al., 2009; Zhao et al., 2016). In a lymphoma mouse model, anti-CD47 antibodies prevented lymphoma cell proliferation and prolonged survival (Piccione et al., 2016). Another study also reported that a CD47-blocking antibody led to delayed progression of metastasis and prolonged survival in mice with pancreatic tumours (Cioffi et al., 2015). Several CD47-targeting antibodies or drugs have reached clinical trials (Martinez-Torres et al., 2015; Advani et al., 2018; Huang et al., 2018). Recent results from Zhang *et al.* showed that blocking CD47 effectively triggered macrophage phagocytosis and cytotoxicity against non-small-cell lung cancer relapsing from antiangiogenic treatment (Zhang et al., 2019). Xuyao Zhang *et al.* revealed that a fusion protein VEGFR1D2-SIRPαD1 could effectively elicit potent anti-tumor effects for glioblastoma through disrupting angiogenetic axis and CD47-SIRPα anti-phagocytic axis (Zhang et al., 2018). Consistent with these studies, our study showed that anti-CD47 effectively reversed macrophage phagocytic inhibition of antiangiogenic therapy and enhanced antitumour efficacy in gastric tumour models. Previous research has shown increased VEGF-A expression in tumours from CD47 blockade in tumour stromal cells (Gao et al., 2017). Haiqing Ni *et al.* further demonstrated that a blocking anti-CD47 antibody, named IBI188, increased VEGF-A levels in AML xenograft models (Ni et al., 2021). Herein, we confirmed that combined treatment with bevacizumab and anti-CD47 antibody resulted in an enhanced antitumour effect. In clinical practice, pulmonary metastasis is common in patients with gastric cancer and is known to be associated with a poor prognosis in these patients (Shiono et al., 2013; Iijima et al., 2016; Oguri et al., 2019). Herein, we isolated lung tissues from experimental gastric tumour models in Bev plus anti-CD47-treated cells to elucidate tumour metastasis. We revealed, for the first time, that this combined strategy is an effective therapeutic target for tumour suppressors by inhibiting tumour metastasis.

There were some limitations to this research. First, mechanism by which HIF and CD47 axis became activated on gastric cancer cells relapsing from anti-angiogenic therapy is quite preliminary. More test should be performed to elucidate the underlying mechanism in the future. Second, VEGF is a potent immunomodulator acting in part through direct effects on T cells, a xenograft model has limited predictive power to evaluate how anti-VEGF/CD47 therapeutic combinations would regulate tumor growth in immune competent mice or humans.

## Conclusion

In conclusion, our study demonstrated the upregulation of the innate immune checkpoint CD47 for immunosuppression by increasing HIF-1 expression during bevacizumab therapy. Synergistic therapy targeting CD47 and VEGF reversed macrophage migration and phagocytosis, which were inhibited by bevacizumab therapy and enhanced antitumour effects. Furthermore, blockade of CD47 induced by bevacizumab therapy inhibited tumour metastasis. These results provide an effective strategy for resistance to antiangiogenic therapy, which promote clinical cancer treatment with antiangiogenic drugs in combination with CD47-targeting, may be a feasible therapeutic strategy for gastric cancer treatment.

## Data Availability

The original contributions presented in the study are included in the article/Supplementary Material, further inquiries can be directed to the corresponding authors.

## Abbreviations

VEGF, vascular endothelial growth factor; CD47, cluster of differentiation 47; SIRPα, signal regulatory protein alpha; FDA, Federal Food and Drug Administration; HIF-1, hypoxia-inducible Factor 1; GFP, Green fluorescent protein; qPCR, quantitative polymerase chain reaction; PE, Phycoerythrin; Ab, ntibody; CD31, platelet endothelial cell adhesion molecule-1; PBS, hosphate buffered solution.
