# Supplementary figures and images for: Blocking CD47 Shows Superior Anti-tumor Therapeutic Effects of Bevacizumab in Gastric Cancer
Source: Front Pharmacol. 2022 May 25;13:880139. doi: 10.3389/fphar.2022.880139 (PMC9175199; doi:10.3389/fphar.2022.880139)

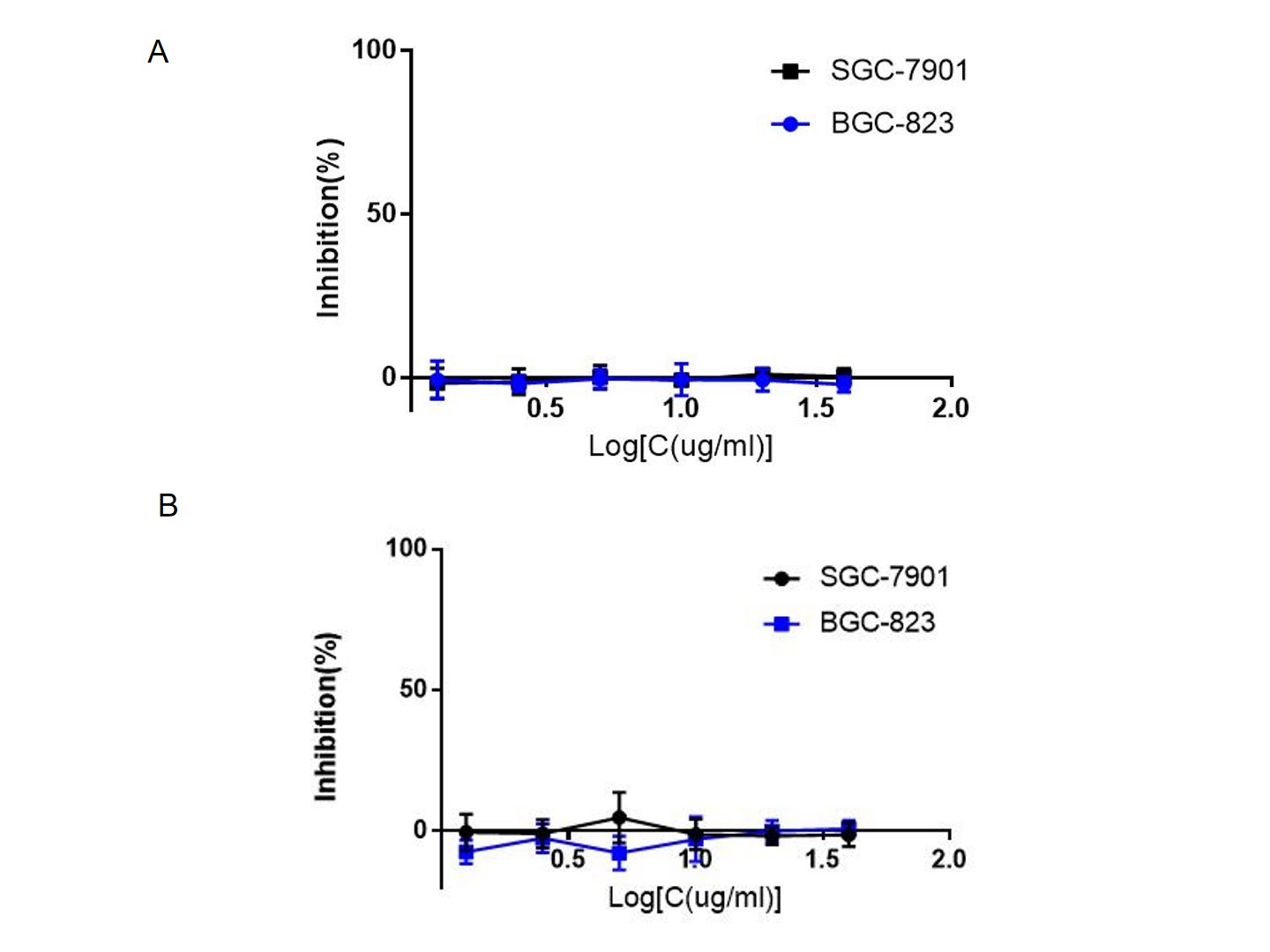

Supplement: Supplementary file 1 [file Image3.JPEG]

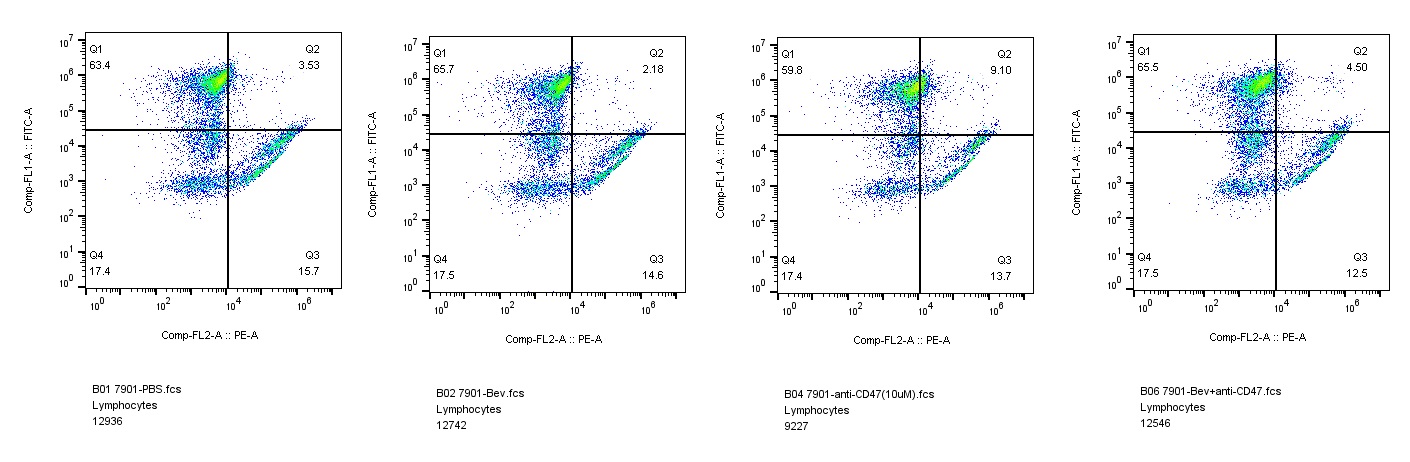

Supplement: Supplementary file 4 [file Image1.JPEG]

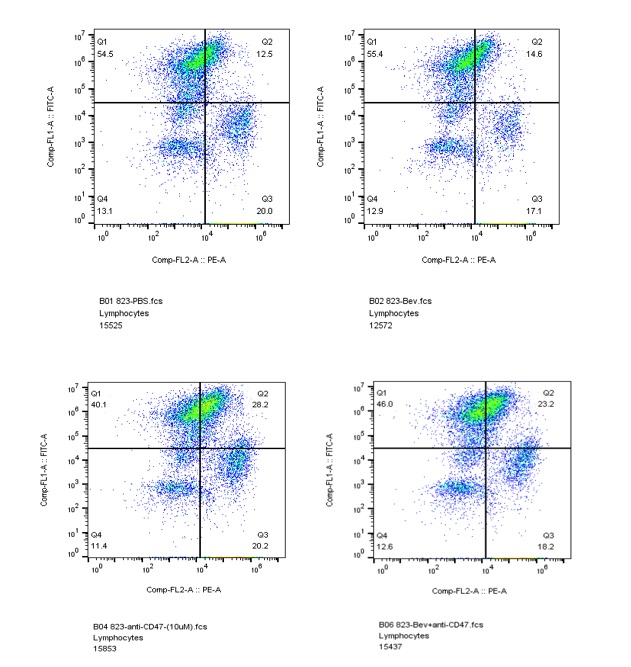

Supplement: Supplementary file 6 [file Image2.JPEG]
