# Supplementary material for: Blocking CD47 Shows Superior Anti-tumor Therapeutic Effects of Bevacizumab in Gastric Cancer
Source: Front Pharmacol. 2022 May 25;13:880139. doi: 10.3389/fphar.2022.880139 (PMC9175199; doi:10.3389/fphar.2022.880139)
Supplement: Supplementary file 2 [file Table1.docx]

| Cells | Control | Bev | Anti-CD47 | Anti-CD47+Bev |
| --- | --- | --- | --- | --- |
| SGC-7901 | 3.51±0.38 | 2.35±0.19 | 9.75±0.56 | 4.54±0.26 |
| BGC-823 | 12.57±0.50 | 13.93±0.70 | 28.63±0.45 | 22.90±0.44 |

Table 1. Fig. 1A-1B Phagocytic index(%)
