# Supplementary material for: Blocking CD47 Shows Superior Anti-tumor Therapeutic Effects of Bevacizumab in Gastric Cancer
Source: Front Pharmacol. 2022 May 25;13:880139. doi: 10.3389/fphar.2022.880139 (PMC9175199; doi:10.3389/fphar.2022.880139)
Supplement: Supplementary file 3 [file Table8.DOCX]

Table 7. Fig. 3E Body weight (g)

| Days | Body weight (g) | | | | | |
| --- | --- | --- | --- | --- | --- | --- |
|  | PBS | Bev（10mg/kg） | Anti-CD47（10mg/kg） | Bev（10mg/kg）+  Anti-CD47（5mg/kg） | Bev（10mg/kg）+  Anti-CD47（10mg/kg） | Bev（10mg/kg）+  Anti-CD47（20mg/kg） |
| 0 | 17.67±0.75 | 17.50±0.96 | 17.67±0.94 | 16.67±1.25 | 17.67±1.11 | 17.33±0.47 |
| 3 | 18.50±0.76 | 17.50±1.26 | 18.00±0.58 | 18.33±0.94 | 18.17±0.90 | 17.83±0.69 |
| 6 | 18.67±0.75 | 18.17±1.21 | 18.67±1.11 | 18.33±0.94 | 18.50±0.76 | 18.17±0.69 |
| 9 | 18.67±0.75 | 18.17±1.34 | 19.17±1.07 | 18.17±0.69 | 19.17±0.69 | 18.67±0.47 |
| 12 | 19.50±0.50 | 18.50±0.96 | 19.00±0.58 | 18.83±1.57 | 18.83±1.07 | 19.33±0.75 |
| 15 | 18.50±0.76 | 18.83±0.90 | 19.00±0.82 | 18.67±1.11 | 19.33±1.11 | 19.00±1.00 |
| 18 | 18.67±0.47 | 18.00±0.82 | 19.00±1.00 | 18.33±1.89 | 19.17±1.46 | 18.33±0.94 |
| 21 | 18.67±0.75 | 18.17±1.34 | 18.83±1.77 | 18.33±1.25 | 19.50±1.61 | 18.83±0.69 |
| 24 | 19.00±0.58 | 18.67±1.25 | 18.67±1.25 | 18.83±0.69 | 19.50±1.61 | 19.67±1.80 |
