# Supplementary material for: Blocking CD47 Shows Superior Anti-tumor Therapeutic Effects of Bevacizumab in Gastric Cancer
Source: Front Pharmacol. 2022 May 25;13:880139. doi: 10.3389/fphar.2022.880139 (PMC9175199; doi:10.3389/fphar.2022.880139)
Supplement: Supplementary file 5 [file Table7.DOCX]

Table 7. Fig. 3C-2 CD31 positive (%_0_)

| Groups | CD31 positive (%_0_) |
| --- | --- |
| PBS（control） | 7.06±0.76 |
| Bev（10mg/kg） | 2.46±0.09** |
| Anti-CD47（10mg/kg） | 6.52±1.55 |
| Bev（10mg/kg）+ Anti-CD47（5mg/kg） | 2.21±1.17** |
| Bev（10mg/kg）+ Anti-CD47（10mg/kg） | 2.19±0.81** |
| Bev（10mg/kg）+ Anti-CD47（20mg/kg） | 1.59±0.08*** |
| p**<0.01, p***<0.001 vs Control Group |  |
