# Supplementary material for: Blocking CD47 Shows Superior Anti-tumor Therapeutic Effects of Bevacizumab in Gastric Cancer
Source: Front Pharmacol. 2022 May 25;13:880139. doi: 10.3389/fphar.2022.880139 (PMC9175199; doi:10.3389/fphar.2022.880139)
Supplement: Supplementary file 7 [file Table2.docx]

Table 2. Fig. 1C-1D Number of migration cells

| Groups | PBS(control) | Bev | Anti-CD47 | Bev + Anti-CD47 |
| --- | --- | --- | --- | --- |
| Number of cells | 57±4 | 22±6*** | 110±14** | 83±6** |
| **p<0.01, ***p<0.0001 vs Control Group | | | | |
