# Supplementary material for: Blocking CD47 Shows Superior Anti-tumor Therapeutic Effects of Bevacizumab in Gastric Cancer
Source: Front Pharmacol. 2022 May 25;13:880139. doi: 10.3389/fphar.2022.880139 (PMC9175199; doi:10.3389/fphar.2022.880139)
Supplement: Supplementary file 8 [file Table5.DOCX]

Table 5. Fig. 3A-2 Tumour volume (mm^3^)

| Days | Tumour volume（mm^3^） | | |
| --- | --- | --- | --- |
|  | PBS（control） | Bev（10mg/kg） | Anti-CD47（10mg/kg） |
| 0 | 159.73±83.09 | 133.98±36.55 | 129.77±32.00 |
| 3 | 272.88±138.68 | 160.88±53.17 | 218.66±60.63 |
| 6 | 333.03±167.37 | 204.17±71.99 | 290.76±74.74 |
| 9 | 441.52±209.30 | 237.77±66.96 | 389.93±151.66 |
| 12 | 635.52±259.07 | 272.87±61.65* | 487.66±154.27 |
| 15 | 767.31±250.05 | 299.80±42.42** | 454.74±141.91* |
| 18 | 948.64±214.07 | 323.35±78.06*** | 583.57±174.52* |
| 21 | 1077.83±221.94 | 350.40±119.80**** | 742.35±254.62* |
| 24 | 1303.75±347.45 | 492.11±173.67*** | 884.56±230.58* |

| Days | Tumour volume（mm^3^） | | |
| --- | --- | --- | --- |
|  | Bev（10mg/kg）+  Anti-CD47（5mg/kg） | Bev（10mg/kg）+  Anti-CD47（10mg/kg） | Bev（10mg/kg）+  Anti-CD47（20mg/kg） |
| 0 | 130.28±20.17 | 143.64±32.06 | 127.28±43.26 |
| 3 | 160.26±24.88 | 164.44±34.16 | 152.36±55.90 |
| 6 | 144.34±26.10* | 161.28±15.87* | 160.84±53.62 |
| 9 | 176.19±40.30* | 191.93±15.85* | 179.79±60.03* |
| 12 | 165.24±27.59** | 222.30±53.64** | 169.88±48.94** |
| 15 | 209.69±26.00*** | 204.31±38.63*** | 171.84±56.90*** |
| 18 | 210.98±25.71**** | 229.23±49.71**** | 162.94±33.91**** |
| 21 | 278.49±62.67**** | 261.70±72.17**** | 194.72±46.78**** |
| 24 | 296.33±44.86**** | 251.13±65.42**** | 200.24±68.23**** |
| *p<0.05, **p<0.01, ***p<0.001, ****P<0.0001 vs Control Group | | | |
