# Supplementary material for: Blocking CD47 Shows Superior Anti-tumor Therapeutic Effects of Bevacizumab in Gastric Cancer
Source: Front Pharmacol. 2022 May 25;13:880139. doi: 10.3389/fphar.2022.880139 (PMC9175199; doi:10.3389/fphar.2022.880139)
Supplement: Supplementary file 9 [file Table3.DOCX]

Table 3. Fig. 1F CD68 positive(%)

| Groups | | CD68 positive（%） |
| --- | --- | --- |
| PBS（control） | 9.09±0.98 | |
| Bev（10mg/kg） | 4.37±0.15** | |
| Anti-CD47（10mg/kg） | 13.51±1.12* | |
| Bev（10mg/kg）+ Anti-CD47（5mg/kg） | 11.28+0.52* | |
| Bev（10mg/kg）+ Anti-CD47（10mg/kg） | 17.37±0.98** | |
| Bev（10mg/kg）+ Anti-CD47（20mg/kg） | 16.89±1.45** | |
| *p<0.05,**p<0.01 vs control group |  | |
