# Supplementary material for: Blocking CD47 Shows Superior Anti-tumor Therapeutic Effects of Bevacizumab in Gastric Cancer
Source: Front Pharmacol. 2022 May 25;13:880139. doi: 10.3389/fphar.2022.880139 (PMC9175199; doi:10.3389/fphar.2022.880139)
Supplement: Supplementary file 10 [file Table4.DOCX]

Table 4. Fig. 2 Fold of mRNA expression

| Group | mRNA expression | |
| --- | --- | --- |
|  | HIF-1 | CD47 |
| PBS（control） | 1.00±0.04 | 1.02±0.18 |
| Bev（10mg/kg） | 2.31±0.07**** | 1.83±0.21* |
| Anti-CD47（10mg/kg） | 1.39±0.16* | 1.00±0.06 |
| Bev（10mg/kg）+ Anti-CD47（5mg/kg） | 2.11±0.33** | 1.95±0.40* |
| Bev（10mg/kg）+ Anti-CD47（10mg/kg） | 2.36±0.19*** | 1.75±0.09** |
| Bev（10mg/kg）+ Anti-CD47（20mg/kg） | 2.77±0.13**** | 1.81±0.04** |
| *p<0.05, **p<0.01, ***p<0.001, ****p<0.0001 vs control group | | |
