# Supplementary material for: Blocking CD47 Shows Superior Anti-tumor Therapeutic Effects of Bevacizumab in Gastric Cancer
Source: Front Pharmacol. 2022 May 25;13:880139. doi: 10.3389/fphar.2022.880139 (PMC9175199; doi:10.3389/fphar.2022.880139)
Supplement: Supplementary file 11 [file Table6.DOCX]

Table 6. Fig. 3B Tumour inhibition (%)

| Groups | Tumour inhibition（%） |
| --- | --- |
| PBS | - |
| Bev（10mg/kg）（control） | 62.25±13.32 |
| Anti-CD47（10mg/kg） | 32.15±17.69 |
| Bev（10mg/kg）+ Anti-CD47（5mg/kg） | 77.27±3.44* |
| Bev（10mg/kg）+ Anti-CD47（10mg/kg） | 80.74±5.02* |
| Bev（10mg/kg）+ Anti-CD47（20mg/kg） | 84.64±5.23** |
| p*<0.05, p**<0.01 vs Control Group | |
